# Second dose of measles-containing vaccine coverage and associated factors among children aged 24–36 months in Gondar city, Central Gondar, Northwest Ethiopia, 2023

**DOI:** 10.3389/fpubh.2024.1364865

**Published:** 2024-05-02

**Authors:** Molalign Aligaz Adisu, Worknesh Akanaw Bogale, Tewodros Getaneh Alemu

**Affiliations:** ^1^Department of Pediatrics and Child Health Nursing, College of Medicine and Health Sciences, Woldia University, Woldia, Ethiopia; ^2^Department of Pediatrics and Child Health Nursing, College of Medicine and Health Sciences, University of Gondar, Gondar, Ethiopia

**Keywords:** second dose, measles, vaccination, coverage, Gondar, Ethiopia

## Abstract

**Background:**

Measles caused 207,000 deaths worldwide in 2019. Ethiopia ranks among the top 10 countries in the world with the highest number of measles cases. However, the coverage of the second dose of measles-containing vaccine (MCV2) remains low. To increase coverage, the government of Ethiopia launched a nationwide measles vaccination campaign. Despite this intervention, the coverage is still below target, and there is scarce information in the study area. Therefore, this study aimed to assess MCV2 coverage and associated factors among children aged 24–36 months in Gondar city, Central Gondar, Northwest Ethiopia, 2023.

**Methods:**

A community-based cross-sectional study was conducted among 621 children aged 24–36 months using a systematic random sampling technique from 25 April to 25 May. A pre-tested, interviewer-administered, and structured questionnaire was used and collected using Kobo Toolbox and then transferred to Stata version 17 for further analysis. The binary logistic regression model was used to identify factors, and the presence of an association was declared using a *p*-value of <0.05. Similarly, an adjusted odds ratio with a 95% confidence interval was used to interpret the direction and strength of an association.

**Results:**

A total of 621 children, with a response rate of 98.1%, participated in the study. The coverage of the second dose of MCV was 75.68% (95% CI: 72.1–78.9). The following factors were significantly associated with measles-containing vaccine second dose (MCV2) coverage: father as the household head (AOR: 3.06, 95% CI: 1.43–6.44), first birth order (AOR: 4.45, 95% CI: 1.21–16.3), four and above antenatal care (ANC) follow-ups (AOR: 5.18, 95% CI:1.62–16.5), postnatal care (PNC) service utilization (AOR: 2.57, 95% CI:1.27–5.15), at least two doses of vitamin A uptake (AOR: 6.39, 95% CI: 2.67–15.2), mothers having high awareness (AOR: 1.97, 95% CI:1.15–3.4), and good perception (AOR: 3.6, 95% CI: 2–6.47) about measles vaccination.

**Conclusion and recommendations:**

The coverage of MCV2 in the study area is lower than the national and global target of above 95%. Head of household, birth order, ANC follow-up, PNC service utilization, vitamin A uptake, awareness, and perception of mothers about measles vaccination were significant factors for MCV2 coverage. Creating awareness, increasing the perception of mothers about measles vaccination, and strengthening the ANC and PNC services will increase the coverage.

## Introduction

Measles is a highly infectious vaccine-preventable disease caused by the measles virus and characterized by high-grade fever, cough, runny nose, conjunctivitis, and rash (1). It affects all age groups, but children under the age of 5 are highly affected and develop complications such as ear infections, diarrhea, pneumonia, and encephalitis (1, 2). Before the discovery of vaccination, measles was the world’s endemic disease and caused over 30 million cases and 2 million deaths every year in the world (3). From 2000 to 2020, measles vaccination saved over 31.7 million lives worldwide (1), and it decreased the measles mortality rate by 94% in 2020 (4).

Even though safe and cost-effective measles vaccination has been available since 1963, a significant number of people die of measles every year (1). In 2019, there were over 869,000 cases and 207,000 deaths per year globally. From this, almost three-quarters of deaths are among children under the age of 5, and over 90% of them were in low- and middle-income countries (1, 3). Ethiopia has been listed among the top 10 countries globally for measles outbreaks, with monthly reports recording over 3,800 cases in 2022 (5), and among these cases, 79% occurred among unvaccinated populations (6). The case fatality rate of measles in Ethiopia is 3%, and it may reach up to 30% in outbreak time (7).

The World Health Organization (WHO) and United Nations Children’s Fund (UNICEF) recommended the second dose of measles-containing vaccine (MCV2) in 2009, and currently, nations are advised to incorporate MCV2 in their national immunization schedules regardless of the first dose of MCV1 coverage to protect approximately 15% of children who do not develop protective immunity after the first dose (8). Ethiopia launched the MCV2 on 11 February 2019 and administered it to children at the age of 15 months (8, 9).

At the end of 2021, 71% of children were vaccinated for the MCV2 in the world (10). In Africa, the coverage of MCV2 has reduced from 76% in 2012 to 71% in 2019, and further to 61% in 2021 (11). According to the WHO and UNICEF estimations, the MCV2 coverage in Ethiopia in 2019, 2020, and 2021 was 41, 46, and 46%, respectively (12). In addition, the mini–Ethiopian Demographic and Health Survey (EDHS) 2019 report of the 3-month post-introduction of MCV2 uptake was 9.1% (13).

The identified factors that are significantly associated with the coverage of MCV2 were the age of the mother and child, educational status of the mothers, place of residence, mass media accessibility, birth order, vaccine availability, waiting time for vaccination at the hospital, antenatal and postnatal care (PNC) visit, awareness, and perception of mothers about measles vaccination (14–19).

Ethiopia moves to achieve the goal of WHO to control and eradicate measles by 2030 (20). Apart from the introduction of MCV2, the government of Ethiopia has been conducting key strategies such as strengthening routine and supplementary immunizations through the reaching every district (RED) approach and health extension programs with the collaboration of the regional government and other organizations such as WHO, UNICEF, and the Global Alliance for Vaccination and Immunization (GAVI) for the last 3 years (9). Despite significant efforts, the coverage of MCV2 is still low, leading to a measles outbreak reported in 2022 (21). To the best of our knowledge and based on our search, no research has been conducted regarding the coverage of MCV2 in the study area. Across Ethiopia, little is known about the newly introduced MCV2 uptake and the factors associated with it. This study aims to identify the key obstacles that hinder the increase of MCV2 coverage among children and assess coverage and associated factors in the study area.

## Methods

### Study design and setting

A community-based cross-sectional study design was conducted from 25 April to 25 May 2023. The study was conducted in Gondar city. Gondar is located northwest of the Amhara region, approximately 741 km from Addis Ababa, the capital city of Ethiopia. In 2022, the total population of Gondar city was 475,172. Among these, 43,522 were children under 5 years old. It has 1 comprehensive specialized hospital, 1 general hospital, 8 health centers, 14 health posts, and over 50 private clinics. The city is structured into 6 sub-cities, which contain 25 urban and 11 rural kebeles (the smallest administrative unit) (22). All children aged 24–36 months who live in Gondar city were considered the population source, while the study population comprised children aged 24–36 months with their mothers or caretakers in selected kebele of Gondar city. Seriously ill mothers or caretakers who were unable to respond and residents of the study area for less than 6 months were excluded from the study.

### Sample size determination

The sample size was calculated using a single population proportion formula, *n* = Z^2^_α/2_*P (1-P)/d^2^, where *n* is the number of samples required, with the assumptions of a 5% significance level (Z_α/2_ = 1.96), 5% margin of error (d = 0.05), and MCV2 coverage in west Gojjam of 48.1% (*p* = 0.481) from a previous study (23). Considering a design effect of 1.5 and a non-response rate of 10%, the final estimated sample size was:

$\begin{array}{c} n={Z^{2}}_{\alpha /2}P\left(1-P\right)/d^{2}={{\left(1.96\right)}^{2}}^{*}0.481^{*}\left(1-0.481\right)/{\left(0.05\right)}^{2}\\ =383.6^{*}\;1.1^{*}1.5=633 \end{array}$

### Sampling technique and procedure

A stratified multi-stage sampling technique was used. First, 36 kebeles are stratified into 25 urban and 11 rural kebeles. Among these, 6 kebeles from urban areas and 3 kebeles from rural areas (25%) were selected using the lottery method. Next, samples were allocated proportionally to each kebele based on the number of households containing children aged 24–36 months. Households with children aged 24–36 months were selected using a systematic random sampling technique with a “k” value of 6. Public facilities such as churches, mosques, health centers, health posts, or any government offices were used as reference points to select the first household. Households having more than one eligible child, the youngest child was chosen to minimize recall bias. Supplementary Figure S1 shows the sampling techniques used in the study.

### Study variables

The vaccination status of the child (vaccinated/not vaccinated) was the dependent/outcome variable of this study. Socio-demographic factors (child age, maternal age, maternal educational status, marital status, monthly income, family size, mass media access, occupational status of mother, head of household, and residence), maternal and child-related factors (ANC and PNC visits, tetanus toxoid (TT) vaccine uptake, birth order, place of delivery, number of children, MCV1 uptake, pentavalent 3, and vitamin A uptakes), health service- and access-related factors (time taken to reach the nearest facility, waiting time at vaccination center, willingness to open multi-dose vaccine for one or two children, vaccinator, and vaccine availability), awareness, and perception of mothers were the independent variables.

### Operational definitions

#### MCV2 coverage

The proportion of children aged 24–36 months who had received MCV2 in addition to MCV1, regardless of when they received the MCV1, were considered to have received MCV2 and were coded as “1”; otherwise, as “0” (15, 17, 23).

#### Age-appropriate MCV2 coverage

The proportion of children who received MCV2 within 4 days prior to and within 4 weeks after the national recommended age of 15 months in Ethiopia (8, 24).

#### Awareness

Participants who scored above or equal to the median on the seven awareness questions were considered to have a high level of awareness (17, 25).

#### Perception

Seven perception questions were asked, and participants with scores greater than or equal to the mean were considered to have good perception (26).

### Data collection tools and procedures

A structured, interviewer-administered, and pre-tested questionnaire was used to collect the data. The questionnaire was adapted from previous studies after obtaining permission (16, 18, 27, 28). This questionnaire consists of socioeconomic and demographic factors, maternal and childhood vaccination-related factors, health access and service-related questions, and awareness and perception of mothers about measles vaccination-related questions. Information regarding the vaccination status of the child was collected from the vaccination card and mothers/caregivers by asking about the child’s age during vaccination and the site and route of vaccination. Finally, there are options for reasons why mothers do not vaccinate their children. The data were collected by four degree-holder nurses and supervised by two master nurses and the principal investigators.

### Data quality control

The English version of the questionnaire was translated into Amharic and then back into English to preserve consistency, which helped to assure the accuracy of the data. A pre-test was conducted on 5% of the sample (32 participants) from the Ayira kebele, a kebele that was not included in the selected kebeles for the study. After the pre-test, the questionnaires were reviewed and reformatted based on the inputs. The reliability of awareness and perception of mothers about measles vaccination-related questions were evaluated using Cronbach’s alpha (α = 0.77), which is acceptable. The data collectors and supervisors have been given 2 days of intensive training. The data collection process was strictly followed by supervisors and principal investigators every day. Before analysis, data cleaning and cross-checking were conducted, and missing values were treated.

### Data processing and analysis

The data were collected using the Kobo toolbox and exported to Stata version 17 for coding, cleaning, and analysis. Descriptive statistics were used to describe the characteristics of the participants. The chi-square goodness of fit test was conducted for each variable before the regression analysis, and multicollinearity among independent variables was checked using the variance inflation factor (VIF). The mean VIF was found to be 3.33. The Hosmer and Lemeshow goodness of fit test was used to assess the model’s adequacy, yielding a *p*-value of 0.94. Then, significant factors with a *p*-value of ≤0.2 at a CI of 95% in a bivariable analysis were entered into multivariate logistic regression models to control the effect of confounding factors, and the statistical test of association was considered significant at a *p*-value of <0.05. Crude and adjusted odds ratios with their corresponding 95% confidence intervals were computed to see the strength of the association between the outcome and independent variables. Then, the study results were presented in text, figures, and tables.

### Ethical consideration

Ethical clearance was obtained from the ethics review committee of the School of Nursing on behalf of the Institutional Review Board (IRB) of the University of Gondar, with ethical approval number S/N/64/2015, and a letter of permission was obtained from the Gondar city health office. Written informed consent was obtained from each participant before the interview after explaining the purpose of the study. All the information gathered in the study was kept confidential for research purposes only.

## Results

### Socio-economic and demographic characteristics of mother-child pairs

A total of 621 participants, who had children aged 24–36 months were interviewed, with a response rate of 98.1%. The mean age of the mothers was 30.68 (±SD 5.75) years, while the median age of the children was 30 months, with an interquartile range of 27–32 months. Eighty-five percent of the respondents had access to mass media (television, radio, or a smartphone) (Table 1).

**Table 1:** Socio-economic and demographic characteristics of mother-child pairs in Gondar city, Central Gondar, Northwest Ethiopia, 2023 (*N* = 621).

Variable	Categories	*N* (%)	MCV2 status	
			Vaccinated *n* (%)	Not vaccinated *n* (%)
Residence	Urban	565 (90.98%)	430 (76.11%)	135 (23.89%)
	Rural	56 (9.02%)	40 (71.43%)	16 (28.57%)
Sex of child	Male	319 (51.36%)	247 (77.43%)	72 (22.57%)
	Female	302 (48.63%)	223 (73.84%)	79 (26.16%)
Age of mothers (years)	≤25	125 (20.12%)	90 (72%)	35 (28%)
	26–30	184 (29.62%)	152 (82.61%)	32 (17.39%)
	31–35	185 (29.8%)	145 (78.38%)	40 (21.62%)
	≥36	127 (20.45%)	83 (67.35%)	44 (34.65%)
Religion	Orthodox	502 (80.84)	391 (77.89%)	111 (22.11%)
	Muslim	91 (14.65%)	58 (63.74%)	33 (36.26%)
	Protestant	17 (2.7%)	14 (82.35%)	3 (17.65%)
	Catholic	5 (0.8%)	5 (100%)	0 (0%)
	Others	6 (0.97%)	2 (33.33%)	4 (66.67%)
Mother’s educational status	Unable to read and write	61 (9.82%)	31 (50.82%)	30 (49.18%)
	Primary level	168 (27.05%)	119 (70.83%)	49 (29.17%)
	Secondary level	218 (35.1%)	168 (77.06%)	50 (22.94%)
	College and above	174 (28%)	152 (87.36%)	22 (12.64%)
Mother’s occupation	Housewife	352 (56.7%)	253 (71.88%)	99 (28.12)
	Farmer	14 (2.25%)	8 (57.14%)	6 (42.86%)
	Private business	105 (16.9%)	75 (71.43%)	30 (28.57%)
	Government Employee	150 (24.15%)	134 (89.33%)	16 (10.67%)
Marital status	Married	518 (83.41%)	399 (77.03%)	119 (22.97%)
	Others	103 (16.58%)	71 (68.93%)	32 (31.07)
Father’s educational status	Unable to read and write	17 (2.76%)	6 (35.29%)	11 (64.71%)
	Primary level	171 (27.71%)	113 (66.08%)	58 (33.92%)
	Secondary level	198 (32.09%)	156 (78.79%)	42 (21.21%)
	College and above	231 (37.44%)	193 (83.55%)	38 (16.45%)
Father’s occupation	Farmer	54 (8.75%)	34 (62.96%)	20 (37.04%)
	Private business	279 (45.22%)	202 (72.4%)	77 (27.6%)
	Government Employee	222 (35.98%)	191 (86.04%)	31 (13.96%)
	Casual laborer	62 (10.05%)	41 (66.13%)	21 (33.87%)
Head of household	Father	326 (52.5)	241 (73.93%)	85 (26.07%)
	Mother	83 (13.36%)	54 (65.06%)	29 (34.94%)
	Both	201 (32.36%)	169 (84.08%)	32 (15.92%)
	Others	11 (1.78%)	6 (54.54%)	5 (45.45%)
Family size	< 5	509 (81.96%)	393 (77.21%)	116 (22.79%)
	≥ 5	112 (18.04)	77 (68.75%)	35 (31.25%)
Average monthly income (ETB)	< 5,000	152 (24.47)	100 (65.79%)	52 (3.21%)
	5,000–10,000	269 (43.32)	204 (75.84%)	65 (24.16%)
	>10,000	200 (32.21%)	166 (83%)	34 (17%)
Mass media availability	Yes	529 (85.19%)	414 (78.26%)	115 (21.74%)
	No	92 (14.81)	56 (60.87%)	36 (39.13%)

### Maternal and childhood vaccination-related factors

Four hundred eighty-three (77.78%) of the mothers were vaccinated against TT at least once during the pregnancy of the index child. The basic childhood vaccination status of the children was BCG 606 (97.61%), Penta3 609 (98.07%), and MCV1 549(88.41). Forty-four (7%) of the children had never taken Vitamin A in their lives (Table 2).

**Table 2:** Maternal and childhood vaccination status of the children in Gondar city, Central Gondar, Northwest Ethiopia, 2023 (*N* = 621).

Variables	Categories	*N* (%)	MCV2 status	
			Vaccinated *n* (%)	Not vaccinated *n* (%)
Parity	One	148 (23.83%)	116 (78.38%)	32 (21.62%)
	Two	251 (40.41%)	196 (78.09%)	55 (21.91%)
	Three and above	222 (35.75%)	158 (71.17%)	64 (28.83%)
Birth order	First birth order	188 (30.27%)	152 (80.85%)	36 (19.15%)
	Second–fourth birth order	412 (66.35%)	308 (74.76%)	104 (25.24%)
	≥ Fifth birth order	21 (3.38%)	10 (47.62%)	11 (52.38%)
Pregnancy status	Planned	466 (75%)	370 (79.4%)	96 (20.60%)
	Unplanned	155 (25%)	100 (64.52%)	55 (34.58%)
The child lives with whom	Both parents	508 (81.81%)	398 (78.35%)	110 (21.65%)
	Mothers only	87 (14%)	57 (65.52%)	30 (34.48%)
	Fathers only	15 (2.42%)	9 (60%)	6 (40%)
	Others	11 (1.77%)	6 (54.54%)	5 (45.45%)
TT vaccine status	Not received	138 (22.22%)	82 (59.42%)	56 (40.58%)
	At least once	483 (77.78)	388 (80.33%)	95 (19.67%)
BCG	Vaccinated	606 (97.58%)	462 (76.24%)	144 (23.76%)
	No vaccinated	15 (2.42%)	8 (53.33)	7 (46.67%)
Penta3	Vaccinated	609 (98.07%)	467 (76.68%)	142 (23.32%)
	Not vaccinated	12 (1.93%)	3 (25%)	9 (75%)
MCV1	Vaccinated	549 (88.41)	470 (85.61%)	79 (14.39%)
	Not vaccinated	72 (11.59%)	0 (0%)	72 (100%)
Vitamin A	No dose received	44 (7.09%)	12 (27.27%)	32 (72.73%)
	One dose received	147 (23.67%)	100 (68.03%)	47 (331.97%)
	At least two doses	470 (69.24%)	358 (76.17%)	72 (23.83%)

### Health service and access-related factors

Among 621 mothers, two-thirds (65.7%) had four or more ANC visits, and only 149 (24%) used PNC services at least once. The median waiting time for vaccination at the vaccination site was 30 min. One hundred eighty-one (29.2%) of the respondents had a history of postponement or cancelation of their vaccination schedule by vaccinators or health professionals (Table 3).

**Table 3:** Health service and access-related factors to MCV2 among children aged 24–36 months in Gondar city, Central Gondar, Northwest Ethiopia, 2023 (*N* = 621).

Variable	Categories	*N* (%)	MCV2 status	
			Vaccinated *n* (%)	Not vaccinated *n* (%)
ANC follow-up	No follow-up	23 (3.7%)	10 (43.48%)	13 (56.52%)
	1–3 follow-up	190 (30.6%)	119 (62.63%)	71 (37.67%)
	≥ 4 follow-up	408 (65.7%)	341 (83.56%)	67 (16.64%)
PNC service utilization	Not received	472 (76.01%)	334 (70.76%)	138 (29.24%)
	At least once	149 (23.99%)	136 (91.28%)	13 (8.72%)
Place of delivery	Home	8 (1.29%)	1 (12.5%)	7 (87.50%)
	Health center	184 (29.63%)	137 (74.46%)	47 (25.54%)
	Hospital	407 (65.54%)	315 (77.4%)	92 (22.61%)
	Private clinics	22 (3.54%)	17 (77.27%)	5 (22.73%)
Time to arrive at the nearest vaccination center on foot	≤ 30 min	402 (64.73%)	313 (77.86%)	89 (22.14%)
	>30 min	219 (35.27%)	157 (71.69%)	62 (28.31%)
Place of vaccination	Hospital	233 (37.64%)	187 (80.26%)	46 (19.74%)
	Health center	351 (56.7%)	263 (74.93%)	88 (25.07%)
	Health posts	15 (2.42%)	9 (60%)	6 (40%)
	Outreach site	20 (3.23%)	11 (55%)	9 (45%)
Waiting time for vaccination at the vaccination center	≤ 30 min	348 (56.22%)	269 (77.3%)	79 (22.70%)
	>30 min	271 (43.78%)	201 (74.17%)	70 (25.83%)
Ever has been postponed or canceled vaccination schedule	Yes	181 (29.24%)	134 (74.03%)	47 (25.97%)
	No	438 (70.76%)	336 (76.71%)	102 (23.29%)

### Awareness and perception of mothers/caretakers

Among 621 mothers/caretakers, the median score of the participant among seven awareness-related questions about measles vaccinations was 4, with an interquartile range of 2–5. Of all, half of them (51%) had a high awareness of measles vaccination. Regarding the perceptions of the mothers, the mean score was 3.4 with ±1.37SD, and only 263 (42.3%) of them had a good perception of measles vaccination.

### Second dose of measles-containing vaccine coverage

**Figure 1:**
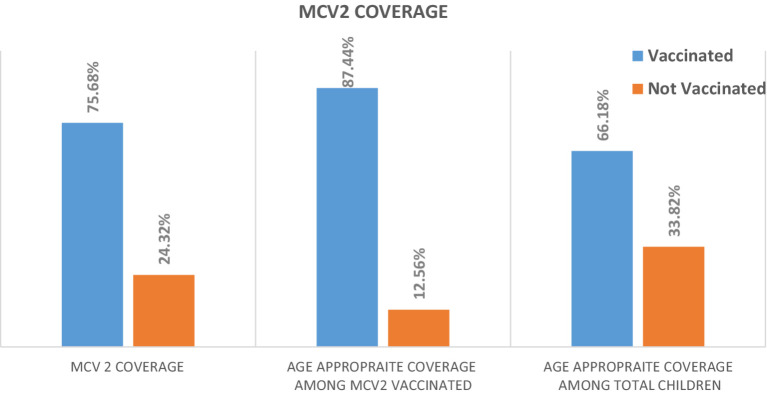
Second dose of measles-containing vaccine coverage among children aged 24–36 months in Gondar city, Central Gondar, Northwest Ethiopia, 2023 (*N* = 621).

Among the 621 surveyed children, 470 (75.68%) (95% CI: 72.1–78.9) were vaccinated for MCV2. The age-appropriate coverage of MCV2 among the total surveyed children was 410 (66.02%) (95% CI: 62.3–69.8), or among those who took MCV2, it was 410 (87.23%) (95% CI, 84.08–90.13) (Figure 1). Thirty-two (5.12%) of the children of mothers/caretakers had no immunization card during the data collection period.

### Reasons for mothers not vaccinating for MCV2

Among 151 unvaccinated children for MCV2, more than half of mothers 83(54.9%) reported being unaware of the need for MCV2, being unable to open the measles vaccine vials for one or two children, and forgetting the schedule (Table 4).

**Table 4:** Frequency of reasons for not vaccinating MCV2 among children aged 24–36 months in Gondar city, Central Gondar, Northwest Ethiopia, 2023 (*N* = 151).

Reasons	Frequency	Percent
Unaware of the need for a second dose of measles vaccination	83	54.90%
Unable to open measles vaccine vial for one or two children	13	8.60%
Forgetting schedule	14	9.27%
Postponed until another time due to unavailable vaccines and vaccinators	12	7.94%
The time of immunization is inconvenient	9	5.96%
Mothers are too busy with many responsibilities	4	2.65%
Family problems, including the illness of the mother	4	2.65%
Lack of faith in immunization	3	1.98%
Child ill	2	1.32%
Not confirmed their vaccination status	7	4.63%
Total	151	100%

### Factors associated with MCV2 coverage

After controlling confounding variables using multivariable logistic regressions: head household, birth order, ANC follow-up, PNC service utilization, vitamin A uptake, awareness, and perception about measles vaccination remained statistically significant factors for MCV2 coverage at a *p*-value of <0.05 with a 95% confidence interval.

Those children whose fathers were the head of the household increased their odds of vaccinating for MCV2 by three times as compared to the children whose mothers were the head of the household (AOR: 3.03, 95% CI: 1.43–6.44). The odds of receiving an MCV2 were 4.45 times higher for children who were born in the first birth order compared to children who were born in birth order five and above (AOR: 4.45, 95% CI: 1.21–16.3). Mothers who had four and above ANC follow-ups increased their odds of vaccinating their children for MCV2 by five times compared to mothers who had no ANC follow-up (AOR: 5.18, 95% CI: 1.61–16.5). Similarly, mothers who had PNC service utilization at least once increased the odds of vaccinating their children for MCV2 by 2.5 times compared to mothers who had not received the PNC service (AOR: 2.57, 95% CI: 1.28–5.15).

The odds of vaccination for MCV2 among children who took vitamin A at least two doses were 6.4 times (AOR: 6.39, 95% CI: 2.70–15.21) higher compared to children who had not received vitamin A. Furthermore, children who received one dose of vitamin A increased their odds of vaccinating for MCV2 by three times compared to children who never received vitamin A (AOR: 3.08, 95% CI: 1.28–7.37). Mothers who had high awareness regarding measles vaccination increased their odds of vaccinating their children for MCV2 by two times compared to those who had low awareness (AOR: 1.97, 95% CI: 1.15–3.40). Similarly, the odds of vaccinating their children for MCV2 were 3.6 times (AOR: 3.60, 95% CI: 2.0–6.5) higher among children of mothers who had a good perception of measles vaccination compared to mothers with a poor perception (Table 5).

**Table 5:** Factors associated with MCV2 coverage among children aged 24–36 months in Gondar city, Central Gondar, Northwest Ethiopia, 2023 (*N* = 621).

Variable	Categories	MCV2 status		COR (95% CI)	AOR (95% CI)
		Vaccinated	Not vaccinated		
Age of mothers	≤25	90 (72%)	35 (28%)	1.36(0.79–2.32)	0.48 (0.2–1.18)
	26–30	152 (82.61%)	32 (17.39%)	2.51 (1.48–4.26)	1.28 (0.61–2.67)
	31–35	145 (78.38%)	40 (21.62%)	1.92 (1.15–3.18)	1.47 (0.74–2.94)
	≥36	83 (67.35%)	44 (34.65%)	1	1
Educational status of mothers	Unable to read and write	31 (50.82%)	30 (49.18%)	1	1
	Primary level	119 (70.83%)	49 (29.17%)	2.35 (1.28–4.29)	0.95 (0.41–2.24)
	Secondary level	168 (77.06%)	50 (22.94%)	3.25 (1.79–5.88)	0.92 (0.35–2.39)
	College and above	152 (87.36%)	22 (12.64%)	6.68 (3.4–13.09)	0.80 (0.23–2.75)
Mother’s occupation	Housewife	253 (71.88%)	99 (28.12)	1	1
	Farmer	8 (57.14%)	6 (42.86%)	0.52(0.176–1.54)	0.83 (0.18–3.79)
	Private business	75 (71.43%)	30 (28.57%)	0.97 (0.60–1.58)	0.84 (0.42–1.67)
	Government Employee	134 (89.33%)	16 (10.67%)	3.27 (1.85–5.78)	1.87 (0.72–4.88)
Father’s occupation	Farmer	34 (62.96%)	20 (37.04%)	0.87 (0.4–1.86)	1.36 (0.48–3.84)
	Private business	202 (72.4%)	77 (27.6%)	1.34 (0.74–2.41)	0.54 (0.23–1.27)
	Government Employee	191 (86.04%)	31 (13.96%)	3.15 (1.65–6.03)	0.84 (0.33–2.13)
	Casual laborer	41 (66.13%)	21 (33.87%)	1	1
Household head	Mother	54 (65.06%)	29 (34.94%)	1	1
	Father	241 (73.93%)	85 (26.07%)	1.52 (0.91–2.54)	**3.03 (1.43–6.44)** ^**^
	Both	169 (84.08%)	32 (15.92%)	2.83 (1.57–5.12)	2.00 (0.91–4.36)
	Others	6 (54.54%)	5 (45.45%)	0.64 (0.18–2.29)	2.36 (0.42–13.20)
Mass media availability	Yes	414 (78.26%)	115 (21.74%)	2.31 (1.45–3.69)	0.75 (0.35–1.58)
	No	56 (60.87%)	36 (39.13%)	1	1
Average monthly income ETB	<5,000	100 (65.79%)	52 (3.21%)	1	1
	5,000–10,000	204 (75.84%)	65 (24.16%)	1.63 (1.05–2.52)	1.05 (0.54–2.06)
	>10,000	166 (83%)	34 (17%)	2.53 (1.54–4.17)	1.11 (0.51–2.43)
Birth order	First birth order	152 (80.85%)	36 (19.15%)	4.64 (1.8–11.77)	**4.45 (1.21–16.3)** ^*^
	Second–fourth order	308 (74.76%)	104 (25.24%)	3.25 (1.34–7.89)	2.03 (0.65–6.30)
	≥Fifth birth order	10 (47.62%)	11 (52.38%)	1	1
Pregnancy status	Planned	370 (79.4%)	96 (20.6%)	2.11 (1.42–3.15)	1.03 (0.59–1.81)
	Unplanned	100 (64.52%)	55 (34.58%)	1	1
ANC follow-up	No follow-up	10 (43.48%)	13 (56.52%)	1	1
	1–3 follow-up	119 (62.63%)	71 (37.67%)	3.61 (1.2–10.82)	2.05 (0.64–6.49)
	≥ 4 follow-up	341 (83.56%)	67 (16.64%)	11.3 (3.8–14.66)	**5.18 (1.62–16.51)** ^**^
TT vaccine status	Not received	82 (59.42%)	56 (40.58%)	1	1
	At least once	388 (80.33%)	95 (19.67%)	2.78 (1.85–4.19)	0.70 (0.38–1.25)
PNC service utilization	Not received	334 (70.76%)	138 (29.24%)	1	
	At least once	136 (91.28%)	13 (8.72%)	4.32 (2.36–7.89)	**2.57 (1.28–5.15)** ^**^
Place of vaccination	Hospital	187 (80.26%)	46 (19.74%)	1	1
	Health centers	263 (74.93%)	88 (25.07%)	0.73 (0.49–1.09)	1.02 (0.61–1.69)
	Health posts	9 (60%)	6 (40%)	0.36 (0.12–1.08)	0.34 (0.09–1.27)
	Outreach site	11 (55%)	9 (45%)	0.3 (0.11–0.768)	0.46 (0.13–1.62)
BCG vaccination	Yes	462 (76.24%)	144 (23.76%)	2.80 (1.01–7.87)	1.52 (0.32–7.10)
	No	8 (53.33)	7 (46.67%)	1	1
Vitamin A uptake	No received	12 (27.27%)	32 (72.73%)	1	1
	1 dose received	100 (68.03%)	47 (331.9%)	5.67 (2.68–11.9)	**3.08 (1.28–7.37)** ^**^
	At least 2 doses	358 (76.17%)	72 (23.83%)	9.25 (6.5–16.9)	**6.39 (2.7–15.21)** ^***^
Level of awareness	Low	200 (64.5%)	110 (35.5%)	1	1
	High	270 (86.82%)	41 (13.18%)	3.62 (2.42–5.41)	**1.97 (1.15–3.42)** ^**^
Level of perception	Poor	231 (64.53%)	127 (35.47)	1	1
	Good	239 (85.28%)	24 (14.72%)	5.47 (3.41–8.77)	**3.60 (2.03–6.47)** ^***^

^*^ *p*-value < 0.05, ^**^ *p*-value < 0.01, and ^***^ *p*-value < 0.005. Bold value means statistically significant variables.

## Discussion

The goal of WHO by the end of 2030 is to make the world free from measles and rubella. To achieve this goal, it is recommended that countries reach above 95% coverage for both the first and second doses of measles vaccination to develop herd immunity (20). However, in Ethiopia, coverage is still below the target (11).

According to the result of our study, the overall coverage of MCV2 among children aged 24–36 months in Gondar city was 75.68% (95% CI: 72.1–78.9). This result is consistent with studies conducted in Malawi (77%) (29) and Zimbabwe (74%) (11). The result of this study is lower than studies conducted in China (93.9%) (28) and Japan (91%) (30). In China, the discrepancy may be due to the difference in awareness and perception of mothers toward MCV2 due to the year of the introduction of MCV2 to routine vaccinations (2005 in China versus 2019 in Ethiopia), the difference in health service accessibility, and sociodemographic characteristics such as the education status of mothers, employment status, and monthly income (28). In Japan, the difference may be because children of mothers who missed the schedule of vaccination were notified by email and letters, and another difference may be the study area; they included participants only from urban settings (30).

However, the result of the study is higher than the studies conducted in Indonesia (54%) (31), Kenya (56.2%) (32), North Shewa, Ethiopia (42.5%) (17), and Jabitehnan district, Ethiopia (48.1%) (23). For the study conducted in Kenya, the possible reason may be due to the difference in the study population (they included children aged 24–59 months, whereas, for this study, the ages included were 24–36 months). The reason for differences from the Indonesian study may be due to the data type they used (demographic health survey data), the study population they included (12–23 months of age), and the study period they conducted (2017). The discrepancy in a study conducted in Shewa and Jabitehnan districts, Ethiopia, may be due to the study population included in their sample, as the majority of their participants were from rural areas. This may result in differences in the awareness and perception of mothers and caregivers about the need to vaccinate their children (33, 34).

According to the results of this study, the odds of vaccinating for MCV2 were higher among households headed by fathers. The findings of this study are consistent with studies conducted in Ethiopia and Shinyanga district, Tanzania: MCV2 was lower among children whose household heads were mothers compared to children whose household heads were fathers. This may be due to high workloads, family responsibilities, and the unequal distribution of economic power and decision-making capacity, which are often skewed toward men in many African countries (35, 36). The finding implies that counseling parents on mutual decision-making concerning the vaccination of their children during healthcare visits may help to improve coverage.

The results show that children who were born in the first birth order had a four times higher chance of being vaccinated for MCV2 than those born in the fifth and above birth order. The finding is consistent with studies conducted in Kenya, Tanzania, Cameroon, and Indonesia (15, 34, 35, 37). The possible justification could be that parental attention can be diverted by the presence of multiple responsibilities as the number of children increases. On the other hand, parents who have only one child could have enough time and resources to vaccinate their child (37). This was also confirmed in this study, as one of the reasons cited by mothers for not vaccinating their children with the second dose of measles vaccination was being busy with many responsibilities. However, to contradict this result, a study conducted elsewhere in Ethiopia reported that mothers with higher birth orders have practical knowledge of the benefits of vaccinations from preceding childbirths (16). Educating parents about the importance of MCV2 and the risk of not vaccinating their children during ANC and PNC visits will improve the coverage of measles vaccination, regardless of birth order.

The chance of children receiving MCV2 was higher when their mothers had four or more ANC follow-ups, compared to mothers who had no ANC follow-ups. Similarly, children of mothers who had at least one PNC service utilization were more likely to vaccinate their children for MCV2 than those who did not have PNC service utilization. This finding is in agreement with the studies conducted in Indonesia, Armenia, Myanmar, Nigeria, Cameroon, sub-Saharan African countries, and elsewhere in Ethiopia (33, 34, 36–40). This can be because mothers who have regular contact with healthcare professionals during their ANC and PNC services are more likely to be encouraged to complete their children’s vaccinations. Through these interactions, mothers can obtain additional information about measles vaccinations, which may help build trust with healthcare professionals (36, 40, 41). Furthermore, repeated visits to health institutions for ANC and PNC services could provide the mothers with the experience of seeing other mothers vaccinate their children. This finding may suggest how maternal health service utilization can increase MCV2 uptake in children.

Children who received vitamin A one or more times had a higher chance of being vaccinated for MCV2 than those who did not receive any. This finding is supported by a study conducted in Kenya (15). The possible reason for this finding is that since Vitamin A is given at 6 and 12 months of age, mothers could be alerted to return for the second dose of measles vaccination when their child is 15 months of age (the narrow gap between 12 and 15 months may help to remember easily), and mothers may have received health education in their consecutive vaccination visits. Encouraging mothers during each visit and clearly informing them of the remaining vaccines their child needs, as well as the location and schedule of vaccination, can help ensure that mothers complete their children’s vaccinations.

Children of mothers who had high awareness about measles vaccination were two times more likely to vaccinate their children for the second dose of measles vaccination compared to mothers who had low awareness. This finding is supported by studies conducted in Kenya and Jabitehnan district, Ethiopia (15, 29). The reason is that mothers or caregivers who have a better understanding of vaccine-preventable diseases, the number of doses of measles vaccine, and the schedule of each measles vaccine could be more committed to vaccinating their children (27). Finally, mothers who had a good perception of measles vaccination had a 3-fold increase in their chance of vaccinating their children with MCV2. This could be because if the mother had a good perception of the severity of the measles and the benefits of the measles vaccine, they would have an increased intention of vaccinating their children. This finding implies that providing information about measles vaccination using social media, encouraging them to discuss their children’s vaccination status with their healthcare providers, making it easy for mothers to find information about measles vaccination, using clear and concise language when discussing vaccination, and answering their questions calmly and reassuringly may all help to improve the awareness and perception of mothers.

### Limitations of the study

Some children had no vaccination card; information regarding their vaccination status was limited to their mothers’ responses, which may be liable to recall bias. However, since MCV2 is given separately from other vaccines and is newly introduced, it is easy for mothers to remember the vaccination status of their children. Furthermore, we ask for the age of the child at vaccination time and the route and site of vaccination to confirm whether the child is vaccinated for MCV2 or not.

## Conclusion

The coverage of MCV2 in the study area is low compared to national and global targets of 95%. Household head, birth order, ANC follow-up, PNC service utilization, vitamin A uptake, awareness, and perception of mothers about measles vaccination were statistically significant factors for MCV2 coverage.

### Recommendations

Healthcare providers should improve maternal and child health services by creating awareness about the schedules, places, and number of recommended doses of the measles vaccine. It is advisable to increase the perception of mothers about the severity of measles and the benefits of MCV2. Efforts should be made to reduce possible obstacles such as fear of side reactions and lack of faith in second-dose measles vaccination. It is also important to encourage mothers to vaccinate their children regardless of birth order. Providing adequate information to the mothers at each schedule about the number of visits still needed for the child until they clearly understood the importance of completing the vaccination schedule.

Policymakers should focus on improving access to ANC and PNC services, strengthening health extension programs and the RED approach, increasing staff at vaccination sites, and ensuring the availability of vaccines at each vaccination site. It is better to integrate mobile health technologies into the EPI schedule to solve many problems, such as forgetfulness, vaccine stockouts, and being unable to open measles vaccines for one or two children. We recommended having a vaccination week at least once a year to increase awareness and perception of measles vaccinations for the community and to vaccinate children who missed the vaccinations for different reasons by mobilizing the whole community, religious and community leaders, political authorities, activists, and other non-governmental organizations in mass media and other public conferences.

## Data availability statement

The raw data supporting the conclusions of this article will be made available by the authors, without undue reservation.

## Ethics statement

The studies involving humans were approved by school of nursing on behalf of the Institutional Review Board (IRB) of the University of Gondar. The studies were conducted in accordance with the local legislation and institutional requirements. Written informed consent for participation in this study was provided by the participants’ legal guardians/next of kin.

## Author contributions

MA: Conceptualization, Data curation, Formal analysis, Funding acquisition, Investigation, Methodology, Project administration, Resources, Software, Supervision, Validation, Visualization, Writing – original draft, Writing – review & editing. WB: Conceptualization, Methodology, Validation, Visualization, Writing – review & editing. TA: Investigation, Methodology, Project administration, Resources, Visualization, Writing – review & editing.
