# Supplementary material for: Second dose of measles-containing vaccine coverage and associated factors among children aged 24–36 months in Gondar city, Central Gondar, Northwest Ethiopia, 2023
Source: Front Public Health. 2024 May 2;12:1364865. doi: 10.3389/fpubh.2024.1364865 (PMC11097900; doi:10.3389/fpubh.2024.1364865)
Supplement: Supplementary file 1 [file Table_1.DOCX]

Annexes

**Annex I: Information Sheet and Consent form**

Information Sheet and Consent Form to study second dose of measles-containing vaccination coverage and associated factors among children aged 24–36 months in Gondar city, Central Gondar, Northwest Ethiopia, 2023.

**Information sheet**

Greeting: Good morning/afternoon dear participant! My name is _________________. I am working as a data collector for the study being conducted to assess the second dose of measles-containing coverage and associated factors among children aged 24 -36 months in Gondar city. I kindly request you to lend me your attention to explain the study. The information we collect will help to your city and the government at large to plan health services. Now you and your child are selected for the survey. If you agree to participate in the study, you will be expected to answer some questions and I will see the vaccination certificate of the child. It will take 20 minutes.

**Benefits and risk of the study**: The study has no direct benefit/payments for those study participants but they may be indirectly beneficial if the result will be utilized by planners of Gondar city health office by expanding and implementing second dose of measles-containing vaccination related strategies and by solving the associated factors, which could be very beneficiary for the participants and also for the overall community. Participating in this study will not have any risk or harm.

**Rights of Participants**: You and your child have full right either to participate or decline participation in this study as participant. You may respond to all the questions or you may not answer to questions you don’t want to and you may end the interview at any time you want. You can ask any question which is unclear for you.

**Confidentiality:** Any information forwarded will be kept confidential and names will not be written or specified.

**Person to contact**: If you have any further questions or would like to receive further information about the study, please contact:

Corresponding author: Molalign Aligaz: - Email: [molalignaligaz@gmail.com](mailto:molalignaligaz@gmail.com)

Cell Phone: +2519-42-76-73-33

**Informed consent**

I have read the above information on the topic and objective of the study. The topic and purpose of the study are clear to me. I understand that participation in this study is voluntary and that I am not obliged to continue if I wish to withdraw from it at any time. I understand that there are no risks associated with participating in this study. So, I agree with this. As a result, I was able to provide informed consent to participate in this study.

Participants signature:

**Interviewer**

Name signature

Date of interview

**Result of interview** 1. Completed 2. Partial completed 3. Refused

**Checked by:**

Supervisors Name signature

**Question Code……….**

**Annex II: English Version Questionnaires**

**Part I. Sociodemographic related Questions**

| **101** | Age of the child (in months) | ………………… | | | | |
| --- | --- | --- | --- | --- | --- | --- |
| **102** | Sex of the child | 1. Male 2. Female | | | | |
| **103** | Residence | 1. Urban 2. Rural | | | | |
| **104** | Mother’s Age (in years) | …………………. | | | | |
| **105** | Religion | 1. Orthodox 2. Muslim 3. Protestant 4. Catholic 5. Others | | | | |
| **106** | Maternal education status | 1. Unable to read and write 2. Able to read and write without formal education 3. Primary (1-8) 4. Secondary (9-12) 5. College and above | | | | |
| **107** | Mother’s occupation | 1. Housewife 2. Farmers 3. Business 4. Government professional 5. Casual labourer | | | | |
| **108** | Marital status of mother? | 1. Single 2. Married 3. Separated 4. Divorced 5. Widowed | | | | |
| **109** | Father’s education status | 1. Unable to read and write 2. Able to read and write without formal education 3. Primary (1-8) 4. Secondary (9-12) 5. College and above | | | | |
| **110** | Father’s occupation | 1. Farmers 2. Business 3. Government professional 4. Casual labourer | | | | |
| **111** | What is the average monthly income in ETB? | …………. | | | | |
| **112** | Head of house Hold | 1. Mother 2. Father 3. Others | | | | |
| **113** | Family size | ……….. | | | | |
| **114** | Have you a Television or radio or smart phone? | 1. Yes 2. No | | | | |
| **Part II. Maternal and child related Questions** | | | | | | |
| **201** | Number of parities | …………. | | | | |
| **202** | Order of the index child | …………….. | | | | |
| **203** | With whom does child live? | 1. Both parents 2. Mothers only 3. Fathers only 4. Others (_________) | | | | |
| **204** | Pregnancy status for the index child | 1. Planned 2. Unplanned | | | | |
| **205** | Did the mother attend antenatal care during pregnancy of the current child? | 1. Yes 2. No | | **If No go to Question 207** | | |
| **206** | If you have ANC visit, how many times do you have? | ………….. | | | | |
| **207** | Did you attend PNC visit? | 1. Yes 2. No | | **If ‘No’ go Question 209** | | |
| **208** | If you have PNC visit, how many times do you attend? | ………… | | | | |
| **209** | Did you take the tetanus toxoid vaccine during pregnancy? | 1. Yes 2. No 3. I don’t know | | | | |
| **Part III. Health Service and Access related Questions** | | | | | | |
| **301** | Where was the child delivered? | 1. Home 2. Hospitals 3. Health center 4. Private clinics | | | | |
| **302** | On foot how long do you take to reach the nearest immunization center? | **………….** | | | | |
| **303** | Does your child had vaccinated for any vaccine? | 1. Yes 2. No | **If No go to Question 306** | | | |
| **304** | Where was Place of vaccination? | 1. Hospital 2. Health center 3. Health post 4. Outreach site | | | | |
| **305** | Waiting time for vaccination? | ………….. | | | | |
| **306** | Have you ever turned without getting vaccination? | 1. Yes 2. No | | | | |
| **307** | Have schedules ever been cancelled or postponed? | 1. Yes 2. No | | | | |
| **Part IV. Mother’s awareness related questions** | | | | | | |
| **401** | Is measles being vaccine-preventable disease? | | | | | - 1. Yes 2. No |
| **402** | At what age should the child start vaccination? | | | | | ……………… |
| **403** | At what age should the child finish vaccination? | | | | | ……………… |
| **404** | How often should children get the measles vaccine? | | | | | ………………, |
| **405** | At what age does the child take the first dose measles vaccine? | | | | | ………………. |
| **406** | At what age does the child take second dose measles vaccine? | | | | | ……………… |
| **407** | Does measles Vaccination have free of Charge? | | | | | 1. Yes 2. No 3. I don’t know |
| **Part V: Mothers/caretakers perception related question** | | | | | | |
| **501** | Do you believe measles is contagious? | | | | 1. Yes 2. No 3. I don't know | |
| **502** | Do think measles vaccine prevent measles? | | | | 1. Yes 2. No 3. I don't know | |
| **503** | Do you think Second dose measles vaccination is included in the routine vaccination schedule? | | | | 1. Yes 2. No 3. I don't know | |
| **504** | Do you believe second measles vaccine being important? | | | | 1. Yes 2. No 3. I don’t know | |
| **505** | Do you think mild fever may occur after second dose measles vaccine? | | | | 1. Yes 2. No 3. I don't know | |
| **506** | Do you worried Second dose measles vaccines can cause your child sick? | | | | 1. Yes 2. No | |
| **507** | Do you take your child for vaccination if he/she is sick? | | | | 1. Yes 2. No | |

**Part VI. Child vaccination Status**

Request the mothers/caretakers to bring the child vaccination card and ask the following questions.

| **601** | **Does your child have vaccination card? 1. Yes 2. No** | | |
| --- | --- | --- | --- |
| **602** | **Child Immunization** | | |
|  | Antigen | Status | age at the time of vaccination |
|  |  |  |  |
|  | BCG | 1. Yes 2. No |  |
|  | **MCV2** | **1. Yes 2. No** |  |
|  | MCV1 | 1. Yes 2. No |  |
|  | Penta3 | 1. Yes 2. No |  |
|  | Vitamin A at 6 months | 1. Yes 2. No |  |
|  | Vitamin A at 12 months | 1. Yes 2. No |  |
|  | Vitamin A at 18 months | 1. Yes 2. No |  |
|  | Vitamin A at 24 months | 1. Yes 2. No |  |

**Part VI. Reasons for not vaccinating for MCV2**

**Note:** Ask only one question ‘why the child was not given second dose of measles-containing vaccine and mark appropriately.

| **Reason** | **Mark** |
| --- | --- |
| Unaware of need for second dose measles vaccination |  |
| Place and/or time of immunization unknown |  |
| Fear of side reactions |  |
| forgetting schedule |  |
| Wrong ideas about contraindications |  |
| Postponed until another time |  |
| Lack faith in immunization |  |
| Rumours |  |
| Cultural/ religious reasons |  |
| Place of immunization too far |  |
| Time of immunization inconvenient |  |
| Un able to open measles vaccine vial for one/two child |  |
| Vaccinator absent |  |
| Vaccine not available |  |
| Mother too busy |  |
| Family problem, including illness of Mother |  |
| Child ill |  |
| Long waiting time |  |
| Other _____________________________ |  |

***Thank you very much!!!***

**አማርኛ ቅጅ**

**አባሪ III: የመረጃ እና የፍቃድ ቅጽ**

የሁለተኛ ዙር የኩፍኝ ክትባት ሽፋን እና ከ 24–36 ወር እድሜ ያላቸው ህጻናት ላይ ያለውን ተያያዥ ምክንያቶች ለማጥናት የመረጃ ወረቀት እና የስምምነት ቅጽ በጎንደር ከተማ፣ ማዕከላዊ ጎንደር፣ ሰሜን ምዕራብ ኢትዮጵያ፣ 2023።

**1-ለተሳታፊዎች መረጃ መስጫ**

እንደምን ዋሉ/አደሩ? ስሜ _________________. በጎንደር ከተማ ከ 24–36 ወራት ዕድሜ ክልል ውስጥ የሚገኙ ሕፃናትን ሁለተኛ ዙር የኩፍኝ ክትባት ሽፋን እና ተያያዥ ጉዳዮችን ለመገምገም እየተካሄደ ላለው ጥናት መረጃ ሰብሳቢ ሆኜ እየሰራሁ ነው። ጥናቱን ለማስረዳት ትኩረት እንድትሰጡኝ በአክብሮት እጠይቃለሁ። የምንሰበስበው መረጃ ለከተማዎ እና ለመንግስት በአጠቃላይ የጤና አገልግሎቶችን ለማቀድ ይረዳል። አሁን እርስዎ እና ልጅዎ ለዳሰሳ ጥናቱ ተመርጠዋል። በጥናቱ ላይ ለመሳተፍ ከተስማሙ, አንዳንድ ጥያቄዎችን እንዲመልሱልኝ እና የልጁን የክትባት የምስክር ወረቀት ዕንድአሳዩኝ እጠይቃለሁ።

**የጥናቱ ጥቅምና ስጋት፡-** በዚህ ጥናት ውስጥ ለመሳተፍ ምንም ሊታዩ የሚችሉ አካላዊ፣ ስነ-ልቦናዊ ወይም ማህበራዊ አደጋዎች ወይም ምቾት ማጣት የለም። ሆኖም፣ ይህ ጥናት እስከ 20 ደቂቃ ጊዜዎን ሊወስድ ይችላል። ይህ ጥናት ለምላሾቹ በገንዘብም ሆነ በሌላ መንገድ ቀጥተኛ ጥቅም የለውም። ነገር ግን ውጤቱ በጎንደር ከተማ ጤና ጥበቃ ጽ/ቤት እቅድ አውጪዎች ጥቅም ላይ ከዋለ ሁለተኛ ዙር የኩፍኝ ክትባት ስልቶችን በማስፋፋትና በመተግበር እና በተዘዋዋሪ ሊጠቅሙ ይችላሉ።

**የተሣታፊዎች መብቶች፡** እርስዎ እና ልጅዎ በዚህ ጥናት ውስጥ እንደ ተሳታፊ የመሳተፍ ወይም የመቃወም ሙሉ መብት አላችሁ። ለሁሉም ጥያቄዎች ምላሽ መስጠት ወይም ለማትፈልጋቸው ጥያቄዎች መልስ አለመስጠት እና በፈለጉ ጊዜ ቃለ መጠይቁን ማቆም ይችላሉ። ለእርስዎ ግልጽ ያልሆነ ማንኛውንም ጥያቄ መጠየቅ ይችላሉ።

**ሚስጥራዊነት፡-** ማንኛውም የተላለፈ መረጃ በሚስጥር ይጠበቃል፣ ስሞችም አይጻፉም፣ አይገለጽም።

**የምታነጋግረው ሰው፡** ተጨማሪ ጥያቄዎች ካሎት ወይም ስለ ጥናቱ ተጨማሪ መረጃ መቀበል ከፈለጋችሁ፡ተመራማሪውን በሚከተለው አድራሻ ማግኘት ይችላሉ።

ስልክ ቁጥር: +251942767333 ኢሜይል[:molalignaligaz@gmail.com](mailto:habtamuhurisa15@gmail.com)

**በመረጃ የተደገፈ የስምምነት ቅጽ፦**

ከላይ በተሰጠው የጥናት ርዕስ እና አላማ ላይ ያለውን መረጃ አንብቤአለሁ/አንብቦልኛል። የጥናቱ ርዕስ እና አላማ ግልጽ ሆኖልኛል። በዚህ ጥናት ውስጥ መሳተፍ በፈቃደኝነት እንደሆነ እና በማንኛውም ጊዜ ከእሱ መውጣት ከፈለግኩ ለመቀጠል እንደማይገደድ ተረድቻለሁ. በዚህ ጥናት ውስጥ ከመሳተፍ ጋር የተያያዘ ምንም አይነት ስጋት እንደሌለ ተረድቻለሁ። ስለዚህ, በዚህ እስማማለሁ. በውጤቱም፣ በዚህ ጥናት ለመሳተፍ በመረጃ ላይ የተመሰረተ ፈቃድ መስጠት ችያለሁ።

የተሳታፊ ፊርማ ________________

ቀን ______________

መረጃ ሰብሳቢ-----------------------------------

ፊርማ፡---------------------

**የቃለ መጠይቁ ውጤት**: 1. የተጠናቀቀ 2. በከፊል የተጠናቀቀ 3. ያልተጠናቀቀ

የተረጋገጠው በ፡ የተቆጣጣሪዎች ስም ----------------------------------

ፊርማ-----------------------------

**ለመሳተፍ ፈቃደኛ ስለሆኑ በቅድሚያ አመሰግናለሁ**

**ጥያቄ ኮድ……………**

**አባሪ IV: አማርኛ ቃለ መጠይቅ**

**ክፍል 1: የስነ ሕዝብ ፣** **የማህበራዊ እና እኮኖምያዊ ነገሮች** በተመለከ **የተዘጋጁ ጥያቄዎች**

| **101** | | የሕጻኑ ዕድሜ ምን ያክል ነው | (የተወለደበት ቀን) __/___/__(በወር): __ | | | |  |
| --- | --- | --- | --- | --- | --- | --- | --- |
| **102** | | የልጅዎ ጾታ: | 1. ወንድ 2. ሴት | | | |  |
| **103** | | መኖሪያ | 1. ከተማ 2. ገጠር | | | |  |
| **104** | | የእናት እድሜ | **…………….** | | | |  |
| **105** | | ሃይማኖት | 1. ኦርቶዶክስ 2. ሙስሊም 3. ፕሮቴስታንት 4. ካቶሊክ 5. ሌሎች | | | |  |
| **106** | | የእናት የትምህርት ሁኔታ | 1. ማንበብ እና መጻፍ የማችል 2. ማንበብ እና መጻፍ የምችል 3. የመጀመሪያ ደረጃ(1-8) 4. ሁለተኛ ደረጃ(9-12 5. ኮሌጅና ከዚያ በላይ | | | |  |
| **107** | | የእናት የስራ ሁኔታ | 1. የቤት እመቤት 2. አርሶአደር 3. የንግድ ስራ 4. የመንግስት ሰራተኛ 5. የቀን ሰራተኛ | | | |  |
| **108** | | የእናት የጋብቻ ሁኔታ | 1. ያላገባች 2. ያገባች 3. ተለያይተው የሚኖሩ 4. የተፋታች 5. ባላ የሞተባት | | | |  |
| **109** | | የአባት የትምህርት ሁኔታ | 1. ማንበብ እና መጻፍ የማይችል 2. ማንበብ እና መጻፍ የሚችል 3. የመጀመሪያ ደረጃ(1-8) 4. ሁለተኛ ደረጃ(9-12 5. ኮሌጅና ከዚያ በላይ | | | |  |
| **110** | | የአባት የስራ ሁኔታ | 1. አርሶአደር 2. የንግድ ስራ 3. የመንግስት ሰራተኛ 4. የቀን ሰራተኛ | | | |  |
| **111** | | ወርሃዊ የገቢ መጠን በብር | …………………… | | | |  |
| **112** | | የቤተሰብ ኃላፊ ማን ነው | 1. እናት 2. አባት 3. ሌሎች | | | |  |
| **113** | | የቤተሰብ ብዛት | ……………….. | | | |  |
| **114** | | ቴሌቪዥን: ሬዲዮ ወይም ስማርት ስልክ አለዎት? | 1. አዎ 2. የለኝም | | | |  |
| **ክፍል II. ከእናቶች እና ከህፃናት ጋር የተያያዙ ጥያቄዎች** | | | | | | | |
| **201** | | እናት የወለደቻቸው ልጀች ብዛት | …………………. | | | |  |
| **202** | | ህጻኑ ስንተኛ ልጅዎ ነው? | ………………….. | | | |  |
| **203** | | ልጅ ከማን ጋር ይኖራል? | 1. ሁለቱም ወላጆች 2. ከእናቱ ጋር ብቻ 3. ከአባቱ ጋር ብቻ 4. ሌሎች(________) | | | |  |
| **204** | | የእርግዝና ሁኔታ | 1. የታቀደ እርግዝና 2. ያልታቀደ እርግዝና | | | |  |
| **205** | | እናት የእርግዝና ክትትል አድርገው ነበር? | 1. አዎ 2. አልተከታተሉም | | **መልስዎ“አልተከታተሉም” ከሆነ ወደ ጥያቄ 207 ይሻገሩ** | | |
| **206** | | ለጥያቄ ቁጥር 205 መልስዎ አወን ከሆነ ምን የህል ጊዜ ተከታተሉ? | **………..** | | | | |
| **207** | | የድህረ ወሊድ ክትትል አድርገው ነበር? | 1. አዎ 2. አልተከታተሉም | | **መልስዎ“አልተከታተሉም” ከሆነ ወደ ጥያቄ 209 ይሻገሩ** | | |
| **208** | | ለጥያቄ ቁጥር 207 መልስዎ አወን ከሆነ ምን የህል ጊዜ ተከታተሉ? | ……………… | | | | |
| **209** | | እናት በርግዝና ወቅት የመንጋጋ ቆልፍ ክትባት ወስዳለች? | 1. አዎ 2. አልወሰድኩም 3. አላውቅም | | | | |
| **ክፍል III. የጤና አገልግሎት እና ተደራሽነት ጥያቄዎች** | | | | | | | |
| **301** | | ልጅዎ የት ነው የተወለደው? | | 1. ቤት 2. ጤና ጣቢያ 3. ሆስፒታል 4. የግል ክሊኒኮች | | | |
| **302** | | ከመኖሪያ ቤትዎ እስከ ክትባት ቦታ በእግር ምን ያክል ይወስዳል? | | ………………………. | | | |
| **303** | | ልጅዎ ክትባት ወስዷል? | | 1. አዎ 2. አልወሰደም | | **መልስዎ“**አልወሰደም**” ከሆነ ወደ ጥያቄ 306 ይሻገሩ** | |
| **304** | | የክትባት ቦታ የት ነበር? | | 1. ሆስፒታል 2. ጤና ጣቢያ 3. ጤና ኬላ 4. የማዳረሻ ጣቢያ | | | |
| **305** | | በክትባት ቦታ ላይ ለክትባት ምን ያህል ሰዓት ይጠብቃሉ? | | ………………. | | |  |
| **306** | | በክትባት ማእከል ውስጥ ልጅዎን ሳያስከተቡ ተመልሰው ያውቃሉ? | | 1. አዎ 2. አላውቅም | | |  |
| **307** | | የክትባት መርሃ ግብሮች ተሰርዘው ወይም ለሌላ ጊዜ ተላልፎቦዎት ያውቃል? | | 1. አዎ 2. አያውቅም | | |  |
| **ክፍል IV. ከእናቶች/ተንከባካቢ ስለኩፍኝ ክትባት ያላቸው እውቀት ጋር የተያያዙ ጥያቄዎች** | | | | | | | |
| 401 | ኩፍኝ በክትባት መከላከል የሚችል በሽታ ነው? | | | | 1. አዎ 2. አይደለም 3. አላውቅም | | |
| 402 | ልጆች በየትኛው ዕድሜ ላይ ነው ክትባት መጀመር ያለባቼው? | | | | …………….. | | |
| 403 | ልጆች በየትኛው ዕድሜ ላይ ነው ክትባቱን ማጠናቀቅ ያለባቼው? | | | | ……………. | | |
| 404 | ልጆች የኩፍኝ ክትባት መውሰድ ያለባቸው ስንት ጊዜ ነው? | | | | ……………………. | | |
| 405 | የመጀመሪያ ዙር ክትባት መቼ ነው መሰጠት ያለበት | | | | ……………………………….. | | |
| 406 | የሁለተኛ ዙር ክትባት መቼ ነው መሰጠት ያለበት | | | | ……………………………… | | |
| 407 | የኩፍኝ ክትባት አገልግሎት ነጻ ነውን? | | | | 1. አዎ 2. አይደለም 3. አላውቅም | | |
| **ክፍል V. ከእናቶች/ተንከባካቢ ስለኩፍኝ ክትባት ያላቸው ግንዛቤ ጋር የተያያዙ ጥያቄዎች** | | | | | | | |
| 501 | ኩፍኝ ተላላፊ ነው ብለው ያምናሉ? | | | | 1. አዎ 2. አላምንም 3. አላውቅም | | |
| 502 | የኩፍኝ ክትባት ኩፍኝን ይከላከላል ብለው ያስባሉ? | | | | 1. አዎ 2. አላምንም 3. አላውቅም | | |
| 503 | ሁለተኛ ዙር የኩፍኝ ክትባት በመደበኛው የክትባት መርሃ ግብር ውስጥ የተካተተ ይመስላችኋል? | | | | 1. አዎ 2. አይ 3. አላውቅም | | |
| 504 | የኩፍኝ ክትባት አስፈላጊ ነው ብለው ያምናሉ? | | | | 1. አዎ 2. አላምንም 3. አላውቅም | | |
| 505 | ከሁለተኛ ዙር የኩፍኝ ክትባት በኋላ ቀላል ትኩሳት ሊከሰት ይችላል ብለው ያስባሉ? | | | | 1. አዎ 2. አይ 3. አላውቅም | | |
| 506 | ክትባቶች ልጅዎን ሊያሳምሙ ይችላሉ ብለው ያስባሉ? | | | | 1. አዎ 2. አይ | | |
| 507 | ልጅዎ ታሞ ቢሆንም ለክትባት ይወስዳሉ? | | | | 1. አዎ 2. አልወስደውም | | |

**ክፍል VI. የልጁ ክትባት ሁኔታ**

የልጁን የክትባት ካርድ እንዲያመጣ ይጠይቁ እና የሚከተሉትን ጥያቄዎች ይጠይቁ።

| **601** | ልጅዎ የክትባት ካርድ አለው | 1, አዎ 2. አይ | |
| --- | --- | --- | --- |
| **602** | **የህጻናት ክትባት መረጃ** |  | |
|  | የክትባት አይነት | የክትባት ሁኔታ | በክትባት ጊዜ የልጁ ዕድሜ |
|  |  |  |  |
|  | BCG/ቢሲጂ | 1. አዎ 2. አልወሰደም |  |
|  | MCV2/ የኩፍኝ ክትባት 2 | 1. አዎ 2. አልወሰደም |  |
|  | MCV1/የኩፍኝ ክትባት 1 | 1. አዎ 2. አልወሰደም |  |
|  | Penta3/ፔንታቫለንት 3 | 1. አዎ 2. አልወሰደም |  |
|  | ቫይታሚን ኤ 6 ላይ ወራት | 1. አዎ 2. አልወሰደም |  |
|  | ቫይታሚን ኤ 12 ወራት ላይ | 1. አዎ 2. አልወሰደም |  |
|  | ቫይታሚን ኤ18 ወራት ላይ | 1. አዎ 2. አልወሰደም |  |
|  | ቫይታሚን ኤ 24 ወራት ላይ | 1. አዎ 2. አልወሰደም |  |

**ክፍል VI: የሚከተሉት ጥያቄዎች ልጆች ሁለተኛ የኩፈኝ ክትባት (MCV-2) ያልዎሰዱበትን ምክንያት የሚዳስሱ ናቸው::**

**Note:** Ask only one question ‘why the child was not given second dose of measles-containing vaccine and circle appropriately.

| **ክትባቱን ያልዎሰዱበት ምክንያት** |  |
| --- | --- |
| ስለክትባቱ አስፈላጊነት ግንዛቤ አለመኖር |  |
| ለሁለተኛ የኩፍኝ ክትባት ድጋኔ መምጣት እነዳለባቸው አለማወቅ |  |
| ክትባቱ የሚሰጥበትን ቦታ ወይም ጊዜ አለማወቅ |  |
| የክትባቱን የጎንዮሽ ጉዳት መፍራት |  |
| ቀጠሮውን መርሳት |  |
| ክትባት መውሰድ ስለመከልከል(contraindications) የተሳሳተ ግንዛቤ |  |
| ቀጠሮውን ወደሌላ ቀን ስላለፈ |  |
| በክትባቱ እምነት ማጣት |  |
| አሉባልታዎች |  |
| ባህል/ሃይማኖት ስለማይፈቅድ |  |
| የክትባት ቦታ መራቅ |  |
| ክትባቱ የሚሰጥበት ሰአት ምቹ አለመሆን |  |
| ለአንድ/ሁለት ልጅ የኩፍኝ ክትባት ጠርሙዝ መክፈት አለመቻል |  |
| ክትባቱን የሚሰጡት ባለሙያዎች አለመኖር |  |
| ክትባት አለመኖር |  |
| እናት ሳራ ሰለበዛባት |  |
| ቤተሰባዊ ችግር (የእናት መታመም) |  |
| ልጁ ስለታመመ |  |
| ክትባት ቦታ ለረጅም ሰአት መቆየት |  |
| ሌላ ካለ ....................... |  |

**አመሰግናለሁ!!!**
