# Supplementary figures and images for: Second dose of measles-containing vaccine coverage and associated factors among children aged 24–36 months in Gondar city, Central Gondar, Northwest Ethiopia, 2023
Source: Front Public Health. 2024 May 2;12:1364865. doi: 10.3389/fpubh.2024.1364865 (PMC11097900; doi:10.3389/fpubh.2024.1364865)

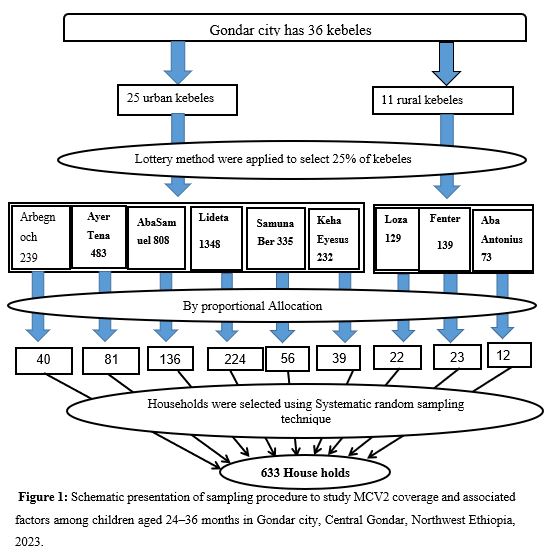

Supplement: Supplementary file 2 [file Image_1.jpg]
